# Effects of Tai Chi on the Executive Function and Physical Fitness of Female Methamphetamine Dependents: A Randomized Controlled Trial

**DOI:** 10.3389/fpsyt.2021.653229

**Published:** 2021-06-10

**Authors:** Shen Menglu, Liu Ruiwen, Yang Suyong, Zhu Dong

**Affiliations:** ^1^Wushu College, Shanghai University of Sport, Shanghai, China; ^2^School of Sport Psychology, Shanghai University of Sport, Shanghai, China; ^3^School of International Education, Shanghai University of Sport, Shanghai, China

**Keywords:** Tai Chi, methamphetamine, executive function, physical fitness, inhibitory control

## Abstract

**Purpose:** Exercise improves the health and mental status of drug dependents. The way by which Tai Chi (TC) as a special exercise treatment affects executive functions (EFs) of methamphetamine (MA) dependents is yet to be established. This study aimed to explore the effects of TC on the EFs and physical fitness of MA dependents.

**Methods:** A total of 76 female MA dependents were randomly assigned to the exercise and control groups. The exercise group underwent three 60-min sessions of TC training per week for 12 weeks. The control group was trained with conventional exercises including the 9th Guang Bo Ti Cao and square dance. Physical fitness and EF assessments that evaluated inhibitory control (IC, go/no-go task), working memory (3-back task) and cognitive flexibility (switching task) were performed at baseline and at 12 weeks. A repeated-measures ANOVA was applied to analyze the differences of group and time.

**Results:** The exercise group showed decreased response time (RT) with a significant main effect of time on the go/no-go task [*F*_(1, 68)_ = 9.6, *p* < 0.05]. The interaction effect between time and group was significant on accuracy [*F*_(1, 61)_ = 4.73, *p* < 0.05], and the main effect of time was significant on RT [*F*_(1, 61)_ = 4.66, *p* < 0.05] in the 3-back task of the exercise group. Significant changes in BMI [*F*_(1, 68)_ = 19.57, *p* < 0.05], vital capacity [*F*_(1, 68)_ = 6.00, *p* < 0.05], and systolic blood pressure [*F*_(1, 68)_ = 6.11, *p* < 0.05] were observed in the exercise group.

**Conclusion:** These findings showed that 3 months of TC training can improve the IC and maintain the working memory and cognitive flexibility of MA dependents. Other data implied that TC may improve the physical fitness of MA dependents.

**Clinical Trial Registration:** http://www.chictr.org.cn/, ChiCTR1900022091.

## Introduction

Methamphetamine (MA) dependence is a growing global concern that has a negative impact on public health (1). MA is reportedly the most widely used illicit drug, second only to cannabis (2). Besides the rapid increase in MA use in Europe (3) and the USA (4), the prevalence of MA use and abuse in Asian has advanced to an alarming degree (5). Approximately 9.9 million of the world's MA users are reportedly living in Asia; and ~1.2 million MA users are residing in China in 2019 (6). Long-term use of MA and other illicit drugs can lead to impairments in the physical health of users (7–10). Several physical fitness aspects of drug dependents (e.g., motor movement, mental status, and cardiopulmonary function) are reportedly poorer than those of normal individuals (11, 12). As a highly addictive psycho-stimulant drug, MA negatively affects individuals' brain structure and function, thus, resulting in behavioral impairments (13, 14). A neuropsychological study found that individuals' cognitive function was significantly impaired after long-term exposure to MA, and such impairment was associated with the abnormal brain function and metabolism due to MA use (15). Compared with dependence to other drugs, MA dependence reportedly causes a more severe impairment in cognitive function (16).

Executive function (EF) is a critical mental process consisting of inhibition, including inhibitory control (IC), working memory, and cognitive flexibility, which are the three core functions of EF (17, 18). EFs are skills essential for mental and physical health (19). Addiction is one of the causes of executive impairments, and similarly, an inseparable relationship exists between poor EF and substance abuse (19). Generally, illicit drug dependents manifest higher impulsiveness in behavior and decision-making than healthy individuals (20–27) due to their impaired EFs. Cognitive studies suggested that individuals who are chronically exposed to illicit drugs have difficulties in execution, inhibition, and decision-making (28). IC is a factor in the prediction of successful recovery in drug dependents (16, 29, 30) and in the maintenance of addiction (31). In executive tasks, MA dependents and other illicit drug users always show poor IC under drug-related stimuli (words or sentences describe MA or scene of MA abuse) (27). Previous studies also found that alcohol, cocaine, nicotine, and Internet dependents have poorer attention span, response time (RT), and accuracy under related stimuli in some EF tasks, indicating their poor IC (32–36). Working memory is the ability to temporarily store the information in the mind; this memory is put to work during the process of executing cognitive tasks (37). A close relationship is reported between working memory and IC; IC is supported by working memory to guide individuals' behaviors to avoid mistakes. When EF is needed, the working memory will shift its function and work together with IC (19). However, drug dependents reportedly have impaired working memory (38, 39). Cognitive flexibility is built on the other two EFs (19), and accumulating evidence indicates that MA dependents exhibit impaired cognitive flexibility (39). The core EFs of the MA dependents are impaired, and the impairment is likely associated, at least in part, with continued drug seeking and using (39).

In recent years, the use of exercise as an intervention to drug dependence has attracted considerable attention (40, 41). Exercise has been proved to greatly ameliorate the EF impairments (42, 43). A systematic review also clarified the positive effects of physical exercise on the enhancement of EF (44). Acute and aerobic exercises were proved to exert positive effects on IC (45–47) according to the results of go/no-go task, which is a psychological task usually used to assess IC (48). Moderate-intensity exercise was also reported to improve working memory performance (49). Moderate-intensity exercise has good effects on EFs because the effect of moderate exercise on IC is superior to other exercises (50, 51). Meanwhile, exercise can improve the health of illicit drug dependents (52). Both short- and long-term moderate-intensity aerobic exercises can enhance their physical fitness and life quality (53). Recent research reported that physical fitness, which includes body mass index (BMI), vital capacity, flexibility, balance and muscle strength, of illicit drug dependents significantly improved after aerobic exercise (54). Withdrawal symptoms of drug users and the likelihood of relapse can also be greatly relieved and reduced by exercises (55, 56).

Tai Chi (TC) is one of the globally renowned Chinese traditional mind–body exercises with a long history. It is the most used intervention method among Chinese tradition exercises because it can mobilize many parts of the body (e.g., dynamic posture control, symmetrical body activities, and body–hand–eye coordination) and reach a certain exercise intensity simultaneously. TC has attracted an increasing amount of attention as a potentially effective method to improve brain health and cognition, and to slow brain aging (57, 58). Several systematic reviews and meta-analysis reported the improved cognition and EF in older adults after TC exercise (59, 60). Studies using brain function test instruments all showed improvements in parameters related to decision-making ability and IC after TC intervention compared with other exercises (61, 62). Nevertheless, studies focusing on the effect of TC on EFs of MA dependents are relatively few. Researchers have focused on the effects of TC on physical and mental health of both male and female drug users, and they reached the consensus that TC exercise has positive influences on mind-body health, specifically on cardiopulmonary function, physical functional performance, and mental status (63–65). A related study provided evidences of the remarkable effects of TC on blood pressure, vital capacity, flexibility, and aerobic endurance in drug users (66). Significant improvements in the physical fitness of hand grip, sit-and-reach, push-up and balance ability, and decreased craving were observed after several months of TC intervention in amphetamine-type dependents (54). Similarly, enhanced life quality of illicit drug users was found following 12 weeks of TC exercise (67). TC also had a beneficial effect on the depression and anxiety symptoms of drug dependents (68). Therefore, TC may be an effective intervention for the physical fitness and EFs of female MA dependents.

Exercise is reportedly beneficial for illicit drug dependents. As a traditional exercise, TC has potential application in the rehabilitation of illicit drug dependents. However, few studies investigated the effects of TC on the EFs of illicit drug dependents. Accordingly, the present study aimed to investigate the effects of TC training on the EFs (IC, working memory, and cognitive flexibility) and physical fitness of female MA dependents. Considering that illicit drug dependents showed improvements in cognition after exercising (41, 69), we hypothesized that TC would exert a positive impact on the EF and improve the physical condition of MA dependents.

## Materials and Methods

### Design

The study is a single-blind randomized controlled trial (ChiCTR1900022091), in which the effect of TC on the EF and physical fitness of MA dependents was examined.

Potential participants were recruited by posting research information in the Shanghai Female Mandatory Detoxification and Rehabilitation Center (SFMDRC). All the participants took part in the study voluntarily. Subjects who participated in the trial and completed the whole study under regulations were rewarded with increased frequency of family calls. Eligible participants were randomized in a 1:1 ratio to the exercise or control group. The randomization sequence was created by SPSS version 25.0 and was unknown to the research staff performing the study procedures. All participants were scheduled to complete a 12-week intervention program post-randomization. The results of EF tasks, including go/no-go, 3-back and switching task, which related to individuals' IC, working memory, and executive control, were measured at baseline and after the intervention. Physical fitness parameters were also assessed simultaneously. The Institutional Review Board of Shanghai University of Sport and Chinese Clinical Trial Register approved the study. Informed consent was obtained from all participants in accordance with the Declaration of Helsinki.

### Participants

**Figure 1:**
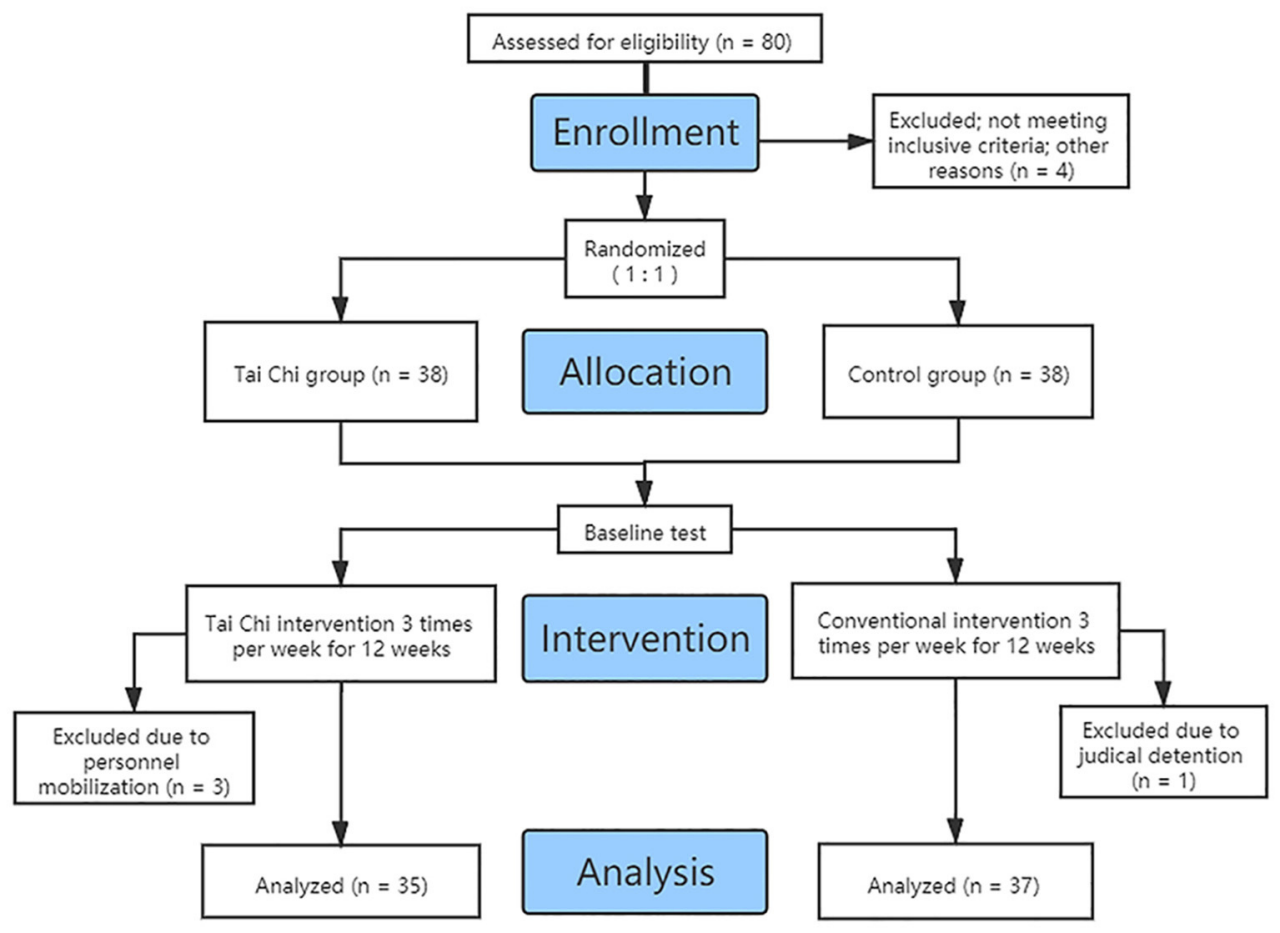
Flowchart of participant enrollment.

The inclusion criteria were as follows: (1) female, aged 18–65 years old; (2) reported history of using MA; (3) no severe medical or mental disease; and (4) educational attainment of primary school or above. The exclusion criteria were as follows: (1) currently diagnosed with Axis I psychiatric disorders, neurological illnesses or trauma affecting the central nervous system; (2) underwent pharmacological treatment with psychotropic medications during or before the study; (3) have anti-social personality disorder and borderline personality disorder; and (4) unwillingness to accept the assigned intervention conditions. Eighty participants were enrolled. Among them, four were excluded for not meeting the criteria. Among these four, three were excluded due to personnel mobilization, and one was excluded due to judicial detention. The final sample comprised 72 participants (Figure 1).

### Physical Fitness Measurements

Blood pressure was measured using OMRON 7113, and the average value was calculated after three consecutive measurements. The maximum lung capacity was recorded after two measurements using an electronic spirometer (FCS-10000, China). A stopwatch was used to measure the length of time the subjects stood on one leg with eyes closed, which reflected their balance ability. Timing was terminated when subjects moved their legs or opened their eyes. The average value was calculated after two measurements. A sit and reach tester was used to measure the flexibility index. The maximum distance was recorded after two measurements. BMI was calculated as weight in kilograms divided by height in meters squared. A grip strength device was used to measure hand grip. Subjects were instructed to grasp the device for a few seconds while exerting their maximum strength. Table 1 summarizes the demographic characteristics and physical fitness parameters of the two groups at baseline.

**Table 1:** Details of the anthropometric information and physical fitness parameters ($\bar{x}$ ± SD).

**Variable**	**Exercise (*n* = 35)**	**Control (*n* = 37)**	***T*-value**	***p*-value**
Age (year)	39.31 ±10.33	39.37 ± 9.28	−0.024	0.981
Height (cm)	159.97 ± 5.96	161.94 ± 5.41	−1.449	0.152
Weight (kg)	61.87 ± 8.18	66.66 ± 9.93	−2.204	0.031^*^
DBP (mmHg)	73.2 ± 10.28	74.29 ± 10.05	−0.447	0.656
SBP (mmHg)	113.74 ± 15.49	109.91 ± 16.16	1.012	0.315
Vital capacity (ml)	1,941.57 ± 551.9	1,978.63 ± 533.7	−0.286	0.776
Flexibility (cm)	11.84 ± 8.76	13.45 ± 6.43	−0.877	0.383
Balance (s)	16.89 ± 12.46	17.71 ± 14.14	−0.260	0.796
BMI (kg/m^2^)	24.13 ± 2.64	25.4 ± 3.46	−1.726	0.089
Hand grip (kg)	26.01 ± 5.81	26.67 ± 5.49	−0.492	0.624

*DBP, diastolic blood pressure; SBP, systolic blood pressure; flexibility, sitting forward bending value; balance, one-leg stand with eyes closed; BMI, body mass index*.

* *Displayed as p < 0.05*.

### Intervention

The exercise group performed a 40 min TC session thrice per week for 12 consecutive weeks. The TC intervention was conducted in the afternoon every Monday, Wednesday, and Friday in the SFMDRC. They were trained in TC, which was based on a simplified 24-form TC that included Ye Ma Feng Zong, Shou Hui Pi Ba, Dao Juan Hong, and other TC styles. Each session consisted of 5 min warm-up, 30 min TC exercise and 5 min cool-down. Participants in the exercise group were instructed by a professional TC instructor two sessions per week. The other session was instructed by a rehab administrator who had been trained for a certain time before the study. The exercise intensity of TC is around 4.5 METs (Metabolic Equivalent of Energy), and individual's heart rate is ~100/beat a session (67).

The control group was trained with conventional exercises, including the 9th Guang Bo Ti Cao and square dance, under the supervision of administrators. The Guang Bo Ti Cao was designed by the China General Administration of Sports, which was divided into eight sections lasting 5 min. The square dance is a moderate-intensity exercise based on the basic movements of aerobics. Each session in the control group involved muscle stretching as warm-up (5 min), square dance and two rounds of Guang Bo Ti Cao as the main intervention (30 min) and cool-down (5 min). The interventions of the two groups were conducted simultaneously in the SFMDRC with short breaks of <60 s and controlled by supervisors.

### Outcome Assessment

#### Primary Outcome Assessment

The go/no-go and switching tasks were selected in the study to assess the core EFs of IC and cognitive flexibility, as they were classic and widely used tasks in psychological studies (19). N-back task, which is often used to assess working memory, was also selected (70). The main analysis was focused on RT and accuracy results. All cognitive tasks were performed manually on a laptop using E-Prime 2.0 software.

##### Go/No-Go Task

**Figure 2:**
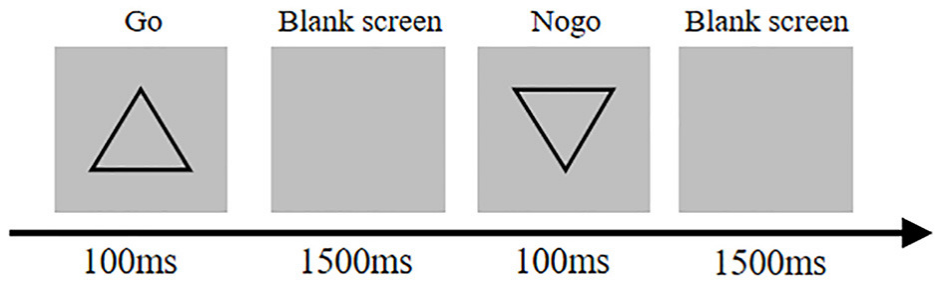
Go/no-go task diagram.

The stimuli were positive (go stimuli) and inverted triangle (no-go stimuli) of the same size (7 × 7 cm) on a gray background placed at the center of the display (brightness set at 60 CD/m^2^). Subjects were instructed to press “F” on the keyboard when the positive triangle appeared as quickly and accurately as possible and to remain seated when the inverted triangle appeared. The stimuli were presented for 100 ms at random order followed by a blank screen with a duration of 1,500 ms. The whole procedure included 20 trials for exercise and 200 trials for the formal task. The accuracy and go RT data were recorded (Figure 2).

##### 3-Back Task

**Figure 3:**
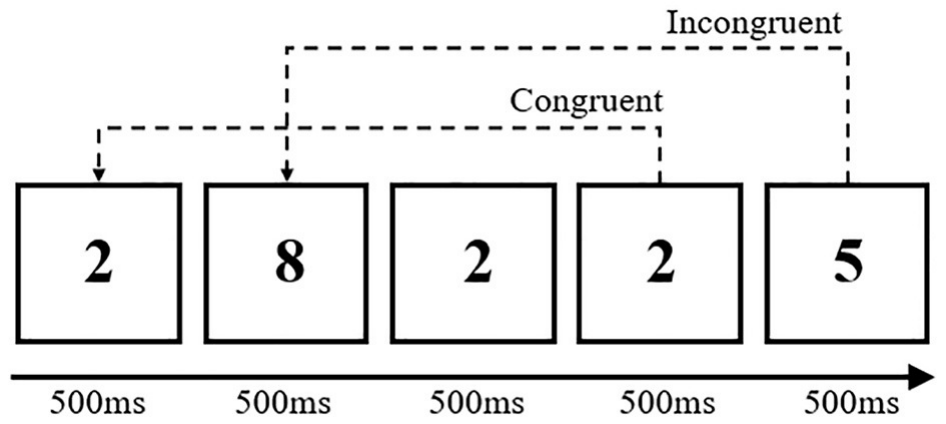
3-back task diagram.

Each trial consisted of a fixation in the center of the screen for 500 ms, followed by 45 numbers presenting continuously for 500 ms with a duration of 2,500 ms. Subjects determined if the presented number matched the next-to-last letter and to respond as quickly and accurately as possible. The task included two conditions, namely, congruent (number matched) and incongruent (number does not match). A short practice trial was given before the formal trial began. The whole procedure comprised 20 trials. The accuracy and RT data were recorded (Figure 3).

##### Switching Task

**Figure 4:**
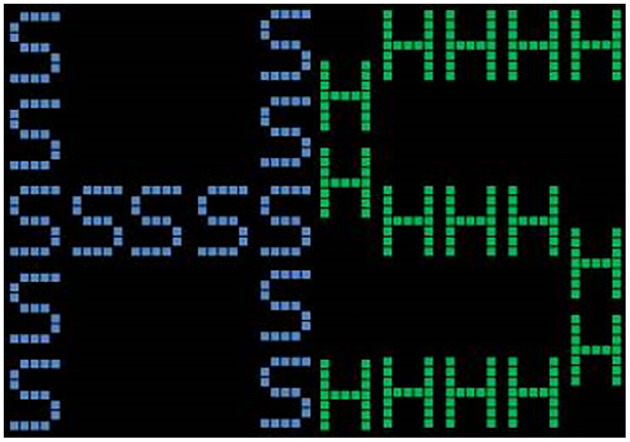
Switching task diagram.

Each trial consisted of a fixation in the center of the screen for 500 ms. This was followed by a stimulus for 500 ms on the screen with an interval duration of 1,000 ms. When the color of the letter turned blue, subjects were instructed to determine whether the whole part (large letter) was S or H and then press the corresponding key (S or H). When the color of the letter appeared green, they were asked to determine whether the part (small letter) was S or H and then press the key (S or H). The formal task included nine sets of stimuli, each comprising 20 trials. The stimuli for each task were presented randomly, and the whole procedure comprised 240 trials. The RT of correct response data were recorded (Figure 4).

### Statistical Analysis

Data were analyzed using SPSS version 25.0. The data for each task were expressed as mean ± SD. Preliminary analyses were conducted to ensure that participant factors did not differ between groups. Univariate analyses also examined whether the observed effects differed depending on participant's characteristics. For the 3-back task, the RTs (congruent and incongruent) and accuracy (congruent and incongruent) were analyzed using 2 (group: exercise vs. control) × 2 (time point: pre-test vs. post-test) repeated measures analysis of variance (ANOVA). The go RT, accuracy (go, no-go) in go/no-go task and RT in switching task were analyzed using repeated measures ANOVA. Physical fitness parameters assessed before and after exercise were analyzed using repeated measures ANOVA. The statistical significance was set at *p* < 0.05.

## Results

### Primary Measurements

#### Go/No-go Task

The analysis of accuracy revealed significant main effects of time [*F*_(1, 68)_ = 9.6, *p* < 0.05, η^2^ = 0.12], condition [*F*_(1, 68)_ = 43.4, *p* < 0.001, η^2^ = 0.39] and the interaction between time and condition [*F*_(1, 68)_ = 20.52, *p* < 0.001, η^2^ = 0.23]. *Post-hoc* test showed that the overall post-test (97 ± 0.3%) accuracy was higher than the pre-test (96 ± 0.2%) accuracy and that the no-go accuracy (95 ± 0.5%) was significantly lower than the go accuracy (99 ± 0.3%). Simple effect analysis showed that the overall pre-test accuracy (94 ± 0.7%) under no-go condition was significantly lower than post-test accuracy (96 ± 0.4%, *p* < 0.01), but the accuracy under the go condition did not differ between time and condition (*p* > 0.05).

**Figure 5:**
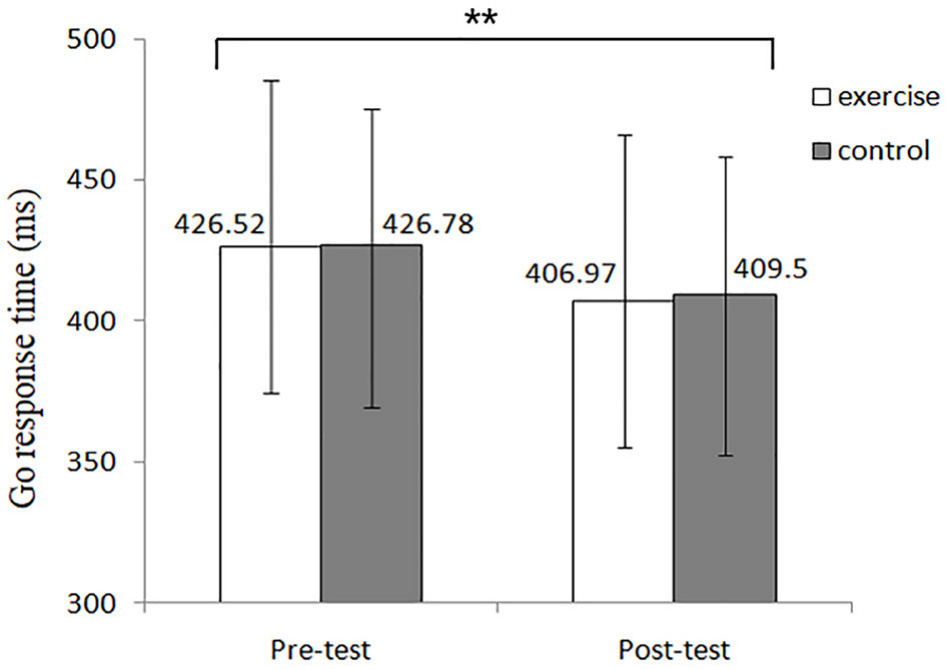
Go response time of the go/no-go task in each group. **Displayed as *p* < 0.01.

RT analysis revealed a significant main effect of time [*F*_(1, 68)_ = 16.86, *p* < 0.001, η^2^ = 0.2]. *Post-hoc* test showed that post-test (408.23 ± 6.52 ms) RT was lower than pre-test (426.65 ± 6.45 ms) RT (Table 2 and Figure 5).

**Table 2:** Behavioral data for the go/no-go task in the exercise and control groups (mean ± SD).

**Condition**	**Exercise (*****n*** **= 35)**		**Control (*****n*** **= 37)**	
	**Pre-test**	**Post-test**	**Pre-test**	**Post-test**
Go accuracy (%)	99 ± 1.0	99 ± 1.0	99 ± 2.0	98 ± 6.0
No-go accuracy (%)	93 ± 6.0	96 ± 4.0	94 ± 5.0	97 ± 4.0

*RT, response time*.

#### 3-Back Task

**Figure 6:**
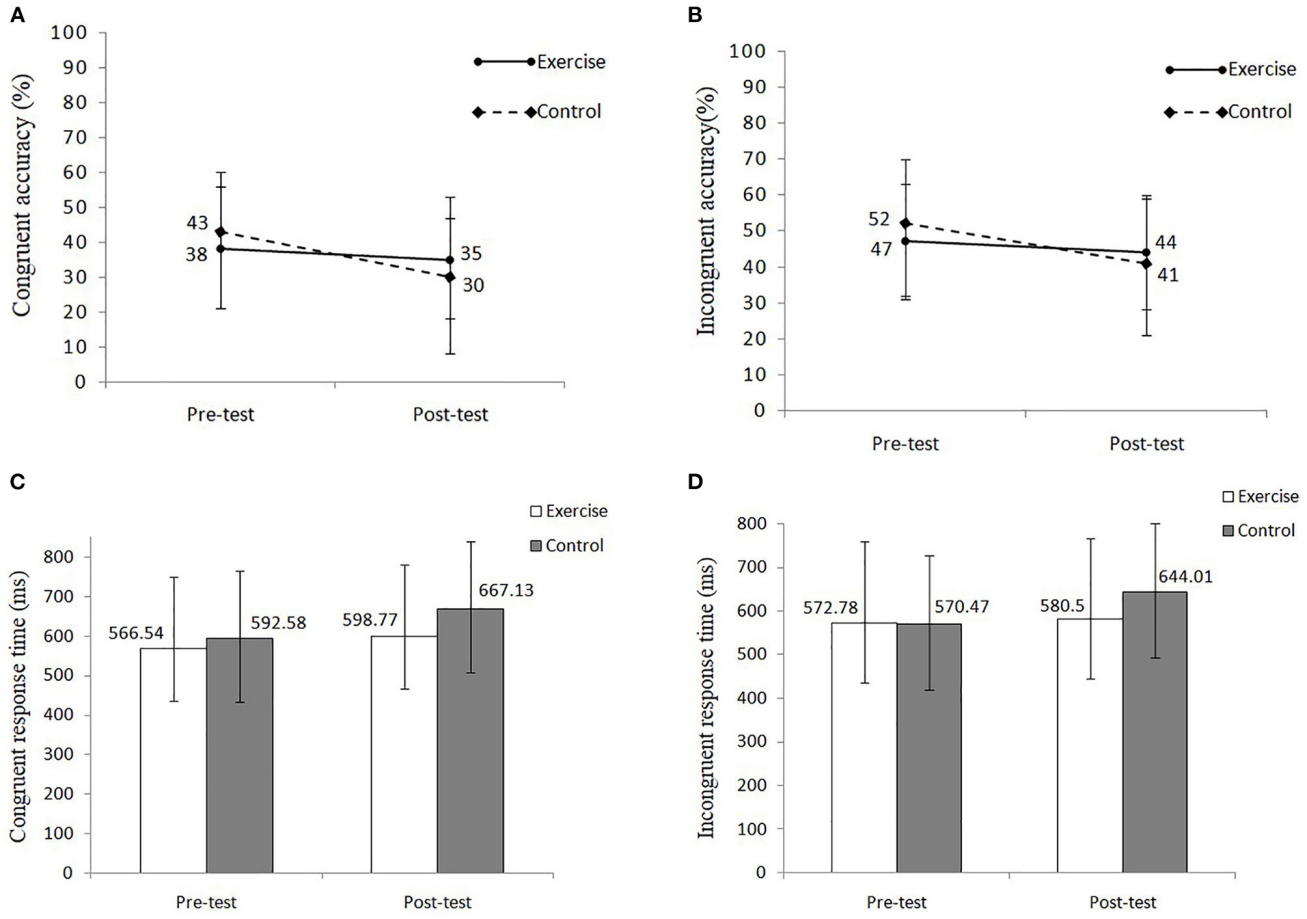
**(A)** Congruent accuracy of the 3-back task in each group. **(B)** Incongruent accuracy of the 3-back task in each group. **(C)** Congruent response time of the 3-back task in each group. **(D)** Incongruent response time of the 3-back task in each group.

The analysis of accuracy showed the significant main effects of time [*F*_(1, 61)_ = 15.43, *p* < 0.001, η^2^ = 0.20], condition [*F*_(1, 61)_ = 17.59, *p* < 0.001, η^2^ = 0.22] and an interaction between time and group [*F*_(1, 61)_ = 4.73, *p* < 0.05, η^2^ = 0.07]. *Post-hoc* test showed that the post-test (37 ± 2.0%) accuracy was lower than the pre-test (45 ± 2.0%) accuracy and that the overall incongruent accuracy (46 ± 2.0%) was significantly higher than the congruent accuracy (36 ± 2.0%). Simple effect analysis showed that the overall post-test accuracy (35 ± 3.0%) of the control group was significantly lower than the pre-test accuracy (47 ± 2.0%) (Figures 6A,B).

The analysis of RT revealed significant main effects of time [*F*_(1, 61)_ = 4.66, *p* < 0.05, η^2^ = 0.07]. *Post-hoc* test showed that the overall post-test RT (622.73 ± 16.45 ms) was significantly higher than the pre-test RT (575.59 ± 20.68 ms) (Figures 6C,D). No other interaction effects were observed.

#### Switching Task

RT analysis showed no significant main effects of time and interaction between time and group (*p* > 0.05) (Table 3).

**Table 3:** RT of switch task in the exercise and control groups (mean ± SD).

**Condition**	**Exercise (*****n*** **= 35)**		**Control (*****n*** **= 37)**	
	**Pre-test**	**Post-test**	**Pre-test**	**Post-test**
RT (ms)	128.58 ± 131.08	129.67 ± 109.21	132.47 ± 140.17	141.82 ± 117.39

*RT, response time*.

### Secondary Measurements

Results of physical fitness parameter analysis indicated significant differences in the within-group factor in terms of systolic blood pressure [*F*_(1, 68)_ = 6.105, *p* < 0.05, η^2^ = 0.082], vital capacity [*F*_(1, 68)_ = 5.998, *p* < 0.05, η^2^ = 0.081] and BMI [*F*_(1, 68)_ = 19.569, *p* < 0.05, η^2^ = 0.223] after 12 weeks of TC intervention. No significant difference was found between groups and interactions of group × time in systolic blood pressure, vital capacity and BMI (Table 4).

**Table 4:** Physical data in the two groups pre- and post-intervention (mean ± SD).

**Variable**	**Exercise (*****n*** **= 35)**		**Control (*****n*** **= 37)**		**Within-group**	**Between-group**	**Time × group interaction**
	**Pre-test**	**Post-test**	**Pre-test**	**Post-test**	***F*-value**	***F*-value**	***F*-value**
DBP (mmHg)	73.2 ± 10.28	71.29 ± 8.1	74.29 ± 10.05	73.69 ± 9.34	1.496	0.745	0.409
SBP (mmHg)	113.74 ± 15.49	105.57 ± 16.07	109.91 ± 16.16	105.14 ± 12.41	6.105^*^	0.732	0.421
Vital capacity (ml)	1,941.57 ± 551.9	2,163.86 ± 635.7	1,978.63 ± 533.7	2,056.46 ± 551.6	5.998^*^	0.084	1.390
Flexibility (cm)	11.84 ± 8.76	12.27 ± 8.53	13.45 ± 6.43	13.63 ± 6.71	0.353	0.717	0.055
Balance (s)	16.89 ± 12.46	18.76 ± 14.13	17.71 ± 14.14	17.84 ± 15.36	0.065	0.318	1.491
BMI (kg/m^2^)	24.13 ± 2.64	23.6 ± 2.57	25.4 ± 3.46	25.08 ± 3.36	19.569^**^	3.676	1.263
Hand grip (kg)	26.01 ± 5.81	26.13 ± 5.2	26.67 ± 5.49	27.18 ± 5.05	0.669	0.483	0.242

*Data were analyzed with repeated measures ANOVA*.

* *Displayed as p < 0.05*.

** *Displayed as p < 0.01*.

*DBP, diastolic blood pressure; SBP, systolic blood pressure; flexibility, sitting forward bending value; balance, one-leg stand with eyes closed; BMI, body mass index*.

## Discussion

The present study provided the initial evidence on how TC training affects the EFs and physical fitness of MA dependents. The provision of a supervised TC training thrice per week for 3 months improved the IC of MA dependents. Meanwhile, their working memory and cognitive flexibility remained stable until the end of the intervention. Positive changes in blood pressure and vital capacity were also observed after 3 months of TC training.

EF deficits, such as response inhibition, decision making, and cognitive flexibility, were found in MA dependents (15, 71). The cognitive performances of illicit drug users can be recovered after a long period of abstinence (72). Meanwhile, exercise can reverse the neurological damage caused by drug dependence (72). The combination of exercise and pharmacological therapy, as an indispensable method of drug withdrawal management, exerted an overall positive effect on drug users (73, 74).

### Executive Function

#### Inhibitory Control

In the go/no-go task, higher accuracy was observed in the exercise group after the TC intervention under no-go condition, whereas lower accuracy was observed in the control group under the go condition in the post-test. Lower RT was found in the exercise group under the go condition in the post-test (Figure 5) compared with the control group.

The positive results in the exercise group were also similar to a previous study which reported that TC intervention can enhance individuals' IC and EF (62). Evidence proved that moderate-intensity aerobic exercise can enhance the executive control ability of both MA addicts and normal individuals (75, 76). TC was considered an integration exercise of aerobic exercise and cognitive training, which may exert more potential effects on executive cognitive ability (62). It was found through a brain research that TC exercise can activate the prefrontal cortex (PFC) and increase oxyhemoglobin and total hemoglobin in this brain region (77). This kind of enhanced brain activation was considered to be correlated with improved cognitive performances (62). Additionally, the effects of TC exercise on mind–body benefits were reportedly more significant than those of regular aerobic exercise (63). A dose–response relationship was also found between the length of TC training and the improvements in EF of individuals. Electrophysiological investigation showed that individuals who consistently participated in TC exercise showed better IC during EF tasks than those in the regular exercise and control groups (61). Therefore, MA dependents may receive facilitated IC through 12 weeks of TC training and may gain benefits from long-term TC training.

#### Working Memory

Although the literature showed evidence that exercise can ameliorate the working memory (78), the present study failed to discover positive changes through the 3-back task. The adverse trend of accuracy and RT in the control group was more obvious than that in the exercise group (Figure 6). Moreover, the exercise group maintained relatively stable levels of accuracy and RT. Previous studies also demonstrated the positive effects of TC on the working memory of normal older population (60). One possible mechanism explained by the researchers was that TC training enhances and activates the brain activity of individuals (77). The movement form of TC is characterized by repetition, similarity, complexity, and diversity. During TC practice, the route and name of each movement needed to be memorized repeatedly, which may require the utilization of the working memory of MA dependents. Therefore, TC training has the potential to stabilize and maintain working memory.

#### Cognitive Flexibility

In previous studies, TC enhances individuals' switching function (79). One research found that individuals' overall cognitive function, processing speed and attention improved after TC intervention (60). Although no significant improvement was found in the present study, the post-test results of the control group showed higher RT compared with baseline and the exercise group, whereas a stable RT was observed in the exercise group. The cognitive flexibility of MA dependents can be well-maintained through TC training.

### Physical Fitness

#### Improvements in Physical Function

Exercise can improve the overall health and basic life quality of MA dependents (80) and is associated with notable improvements in cardiopulmonary health in individuals using MA (52). This study showed significant differences in systolic blood pressure and vital capacity in the exercise group, which was in accordance with many previous studies (81–84). TC exerted influence on the activity of parasympathetic nervous system and the sensitivity of baroreceptor, thereby leading to the positive change of blood pressure (85–87). The vital capacity of the exercise group increased from 1,941.57 ± 551.9 ml to 2,163.86 ± 635.7 ml. This result was similar to a previous study that found MA dependents showed remarkable improvements in vital capacity after moderate-intensity TC intervention (88, 89). TC exercise was also been suggested to normalized BMI in MA users (66). Similarly, decreased BMI was found in the exercise group in the current study, which was the same as the findings of a previous TC intervention study (90).

#### Balance and Flexibility

The duration of one-leg stand with eyes closed increased in the exercise group compared with baseline, which was similar to the finding of another study (54). The fact that TC can improve balance ability for all kinds of people has been proved by many researchers (91, 92). Although no significant differences were found in balance ability, the prolonged standing time indicated that TC had the tendency and potential to improve the balance ability of MA dependents. No significant change in flexibility was observed between the two groups, which was different from the finding that suggest long-term TC practice can increase flexibility (54). However, the improved flexibility found in this study was achieved from 1-year TC intervention, which was longer than the duration used in the current study. Therefore, insufficient duration of intervention may explain the results of our study.

### Possible Mechanism

Numerous studies confirmed the benefits of aerobic exercise on cognitive function (93, 94). The present study showed that TC maintained and improved EFs of MA dependents to some extent. The general mechanism can be explained as follows: Exercise increased the concentration of brain-derived neurotrophic factor (BDNF) (95, 96), which is an important substance that causes neuroplasticity in the brain (96). Exercise training interventions can improve BDNF levels in healthy adults (97, 98), and BDNF has long been implicated in cognition (99). The training-induced BDNF changes reportedly induce cognitive improvement through hippocampal and peripheral levels in humans (99). A neurophysiological study showed that TC improves individuals' memory and other component of EF possibly *via* the upregulation of BDNF (79). Second, MA can cause reduction in striatal and cortical cerebral blood flow *via* dopamine receptor D2 (124) (100), and the blood-brain barrier of individuals can be impaired under the induction of MA (101). However, several studies proved that exercise attenuated MA-induced alterations in neurogenesis and oxidative stress in brain microvessels and protected against blood-brain barrier impairment (102, 103). The neurophysiological effects of exercise to improve blood flow, neurotransmitter levels and brain health have all been proved by evidences (104). This may be the possible mechanism by which TC maintain the EFs of MA dependents in a steadier status. Additionally, as a mind-body exercise, the mindfulness during exercise may influence the brain region involving EFs, such as the anterior cingulate cortex, which is intensely activated during exercise (105). The improvement in EF may due to the neurological flexibility or structural and functional changes in the brain caused by mindfulness practices (106). Additionally, research suggested that long-term TC exercise may contribute to the neural substrates of EF (107).

Therefore, we infer that the improvement of EFs in MA dependents with cognitive deficits may be affected by the combination of the mechanism mentioned above. However, further studies are still necessary to clarify the mechanism.

### Limitations

This study has several limitations. First, participants were all managed by administrators in the drug rehabilitation center. Thus, the blinding of the study may be affected by the relationship between administrator (intervention supervisor) and participants, as they may already know the supervisor. Second, although the general exercise intensity of TC was mentioned in the study, tools or instruments were not used to monitor the exercise intensity of participants in the current study, which may influence the experimental results. Additionally, the use of prolonged intervention duration will add reliability to make up the current study's limitation of having a relative short intervention period. The baseline test on detailed information (e.g., use of drug type, year of drug use, and duration of abstinence before the study) will address the limitation in future studies. The effect of TC exercise on male drug users to enrich the research field can be the focus of future studies, instead of concentrating on females only.

## Conclusion

Three months of moderate-intensity TC exercise improved the IC, maintained working memory and cognitive flexibility of EFs and ameliorated the physical function of female MA dependents. TC was an effective exercise intervention for drug users. However, some changes of EF parameters were not remarkable, which may be due to the short intervention time. Thus, the long-term effects of TC on EF should be explored in the future.

## Data Availability Statement

The raw data supporting the conclusions of this article will be made available by the authors, without undue reservation.

## Ethics Statement

The studies involving human participants were reviewed and approved by The Institutional Review Board of Shanghai University of Sport. The patients/participants provided their written informed consent to participate in this study.

## Author Contributions

SM participated in the design of the study and drafted the manuscript. LR conducted the design of the study, exercise intervention, and partial manuscript writing. ZD participated in the design of this study, coordination of intervention conducted in Shanghai detoxification, and rehabilitation center. YS participated in manuscript revision and study design. All authors read and approved the final manuscript.

## Conflict of Interest

The authors declare that the research was conducted in the absence of any commercial or financial relationships that could be construed as a potential conflict of interest.
